# Synthesis and structure of di­aqua­bis­(nicotinamide-κ*O*)bis­(nitrato-κ^2^*O*,*O*′)calcium(II)

**DOI:** 10.1107/S2056989025006759

**Published:** 2025-08-05

**Authors:** Zulfiya Djumanazarova, Shakhnoza Kadirova, Nuritdin Kattaev, Bakhtiyar Ibragimov, Saule Meldebekova, Jamshid Ashurov

**Affiliations:** a Karakalpak State University named after Berdakh Republic of Karakalpakstan Abdirova Street 1 Nukus 742012 Karakalpakstan; b National University of Uzbekistan named after Mirzo Ulugbek University Street 4 Tashkent 100174 Uzbekistan; c Institute of Bioorganic Chemistry, Academy of Sciences of Uzbekistan, 100125, M., Ulugbek Str 83, Tashkent, Uzbekistan; d Karakalpakstan Medical Institute, 106 A. Dosnazarov Street, 230105 Nukus City, Uzbekistan; University of Aberdeen, United Kingdom

**Keywords:** calcium complex, nicotinamide, hydrogen bonding, crystal structure

## Abstract

The title complex, [Ca(NO_3_)_2_(C_6_H_6_N_2_O)_2_(H_2_O)_2_], crystallizes with an eight-coordinate Ca^2+^ ion in a distorted trigonal–dodeca­hedral coordination environment. The metal ion is coordinated to two nicotinamide ligands *via* their carbonyl O atoms, two bidentate nitrate anions and two water mol­ecules. The nicotinamide ligands adopt a nearly *trans* geometry, while the nitrate anions and aqua ligands are arranged in a pseudo-*trans* fashion. In the crystal, a three-dimensional supra­molecular framework is constructed through N—H⋯O and O—H⋯O hydrogen bonds involving water, nitrate, and nicotinamide functional groups, reinforced by offset π–π stacking inter­actions between nearly parallel pyridine rings [centroid-to-centroid distance = 3.783 (2) Å]. A Hirshfeld surface analysis revealed that the inter­molecular inter­actions are dominated by O⋯H/H⋯O (42.3%) and H⋯H (26.2%) contacts, corresponding to classical hydrogen bonding and van der Waals forces, respectively.

## Abstract 2

The title complex features an eight-coordinate Ca^2+^ center with a distorted trigonal–dodeca­hedral geometry.

## 1. Chemical context

Nicotinamide (niacinamide), a water-soluble form of vitamin B3, plays a pivotal role in human metabolism. It serves as a precursor to the essential coenzymes NAD^+^ and NADP^+^, which are involved in a wide array of redox reactions. NAD^+^/NADH participates in over 400 biochemical processes, while NADP^+^/NADPH is involved in approximately 30 reactions, particularly in cytochrome P450-mediated xenobiotic metabolism (Meyer-Ficca *et al.*, 2016 ▸; Isin *et al.*, 2007 ▸). Beyond its metabolic functions, nicotinamide exhibits versatile coordination behavior due to its ability to donate electron pairs through the pyridine nitro­gen atom and the amide oxygen or nitro­gen atoms. It typically acts as a monodentate ligand *via* the pyridine N atom, but bidentate and bridging coordination modes have also been observed (Pricop *et al.*, 2022 ▸; Sun *et al.*, 2018 ▸). Mixed-ligand complexes involving nicotinamide and 1,10-phenanthroline with Co^II^, Ni^II^, Cu^II^, and Zn^II^ have demonstrated various coordination geometries and potential anti­microbial properties (Drzewiecka *et al.*, 2013 ▸). Similarly, cadmium(II) complexes with nicotinamide, nitrate, and oxalate ligands have shown promising pharmacological activity (Pricop *et al.*, 2025 ▸). In coordination chemistry, the nitrate anion can function as a counter-ion, auxiliary ligand, or redox-active participant. Its inclusion in metal–nicotinamide complexes, such as with calcium(II), may enhance reactivity through NO-release pathways. For instance, recent work by Zhang *et al.* (2024 ▸) shows intra­cellular NO release from nitrate-containing metal complexes, indicating their potential as therapeutic NO donors. Calcium is a biologically essential element involved in diverse physiological roles including bone mineralization, muscle contraction, nerve transmission, and blood coagulation. Emerging research has highlighted calcium’s regulatory function in intra­cellular signaling, gene expression, and metabolic control. Calcium(II)–nicotinamide complexes have garnered inter­est for their structural variety and bioactivity (Braga *et al.*, 2014 ▸; Parsekar *et al.*, 2022 ▸). These include mononuclear species with two nicotinamide and two water ligands and polymeric frameworks where nicotinamide bridges calcium centers (Braga *et al.*, 2011 ▸; Xue *et al.*, 2015 ▸). Mixed-ligand systems incorporating additional donors, such as tri­nitro­phenolates, further demonstrate nicotinamide’s coordination flexibility (Parsekar *et al.*, 2022 ▸). In this study, we report the synthesis and crystal structure of the title complex, [Ca(H_2_O)_2_(C_6_H_6_N_2_O)_2_(NO_3_)_2_], (**I**).
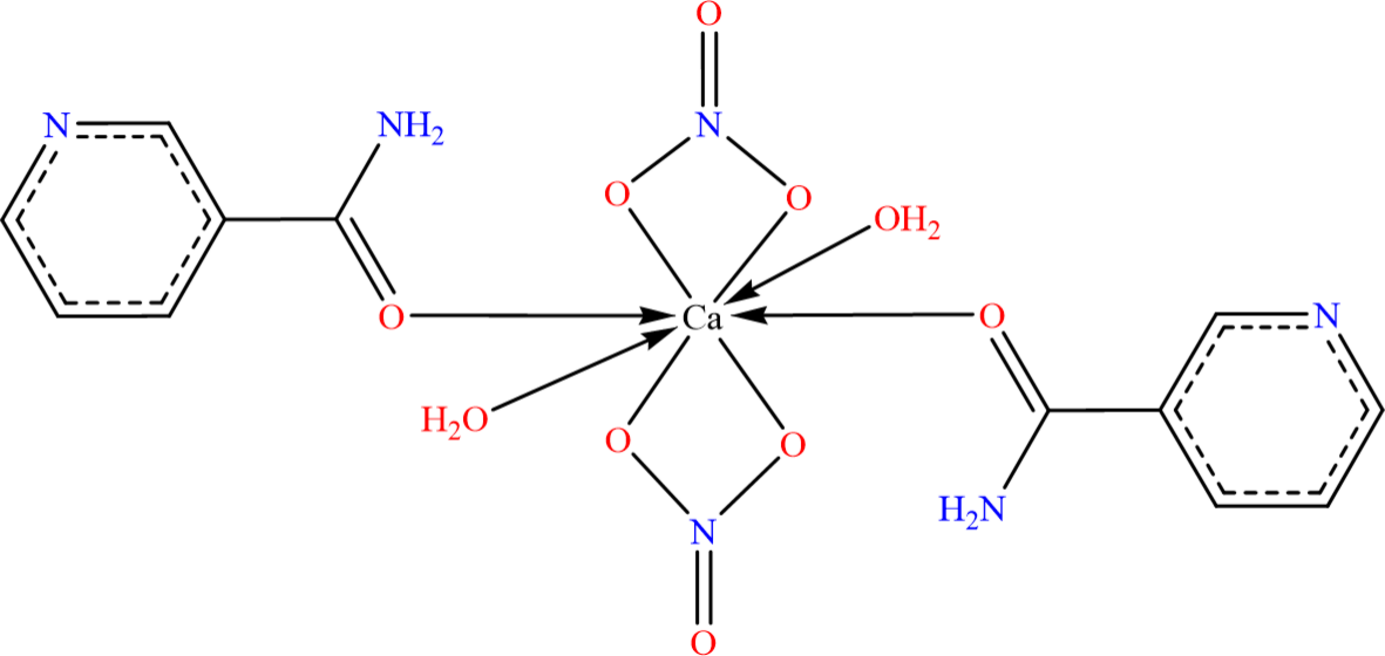


## 2. Structural commentary

**Figure 1:**
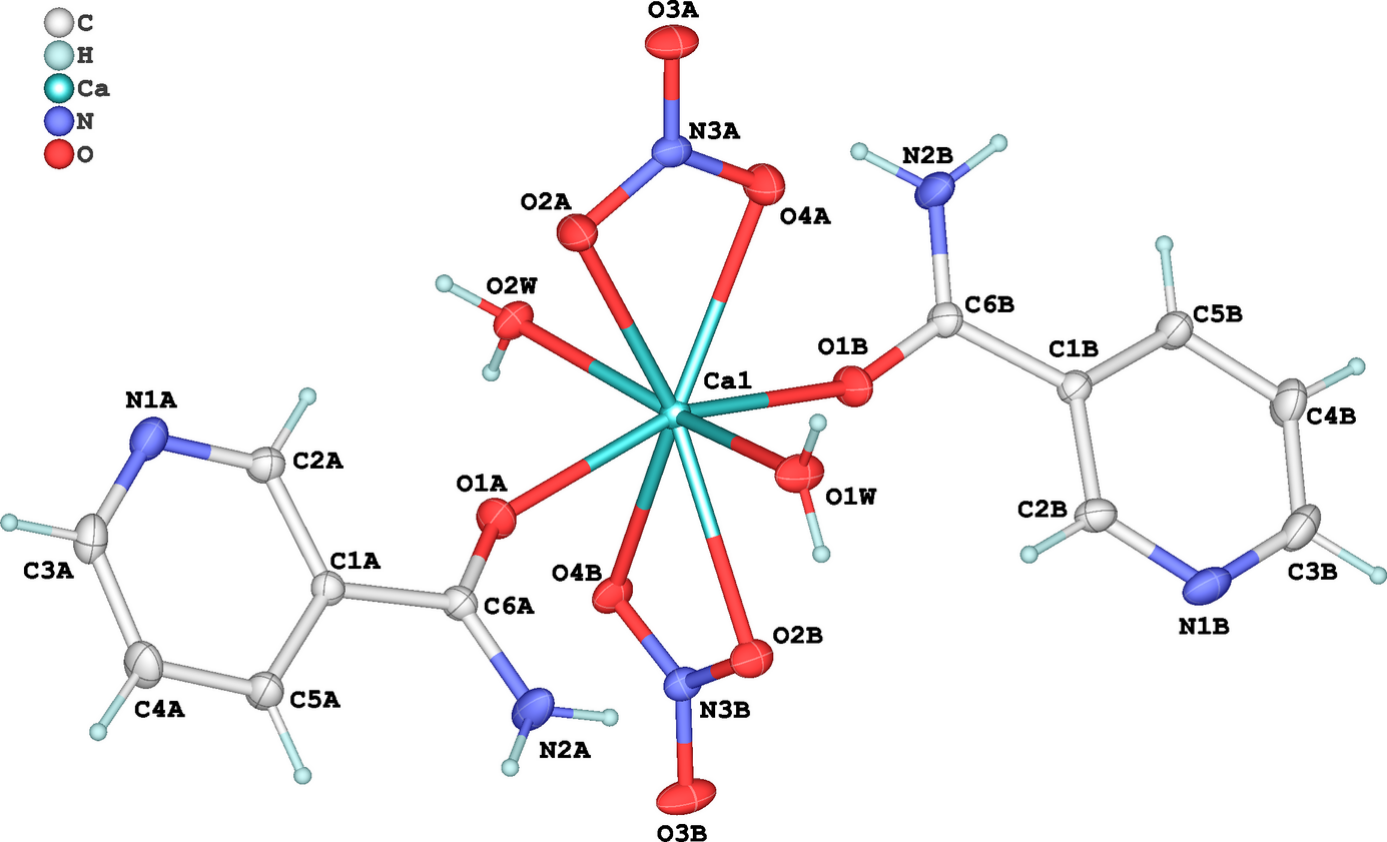
The mol­ecular structure of (**I**) showing 50% probability displacement ellipsoids.

The asymmetric unit of (**I**) contains one calcium(II) cation coordinated to eight oxygen atoms: two from O-monodentate nicotinamide ligands, four from two bidentate nitrate anions, and two from aqua ligands (Fig. 1 ▸). The resulting coordination environment forms a distorted CaO_8_ polyhedron best described as a trigonal dodeca­hedron (also called snub disphenoid). The Ca—O bond lengths (Table 1 ▸) range from 2.3150 (16) Å for Ca1—O1*B* to 2.5825 (18) Å for Ca1—O4*B*. These values are comparable to those reported in a similar Ca^2+^–nicotinamide complex (Xue *et al.*, 2015 ▸), where O-monodentate coordination *via* carbonyl oxygen atoms gave a Ca—O distance of 2.2659 (13) Å, and the Ca—O (water) distance was 2.3774 (11) Å. The nicotinamide ligands in (**I**) are arranged in a nearly *trans* fashion, with an O1*A*—Ca1—O1*B* bond angle of 158.82 (7)°. Similarly, the aqua ligands adopt an approximately *trans* orientation [O1W—Ca1—O2*W* = 164.15 (7)°] and the nitrate oxygen pairs (O2*A*/O2*B* and O4*A*/O4*B*) also exhibit pseudo-*trans* arrangements with angles of 147.25 (6)° and 151.52 (6)°, respectively. Both nicotinamide ligands (mol­ecules *A* and *B*) exhibit the expected near planarity of their aromatic rings, with r.m.s.d. values of 0.003 and 0.002 Å, respectively. The CONH_2_ groups are slightly twisted relative to the pyridine rings, with dihedral angles of 15.37 (12) and 13.33 (12)° for mol­ecules *A* and *B*, respectively. The pyridine ring planes are roughly parallel, forming an inter­planar angle of 7.57 (14)°, whereas the carboxamide planes are more tilted relative to one another, with an inter­planar angle of 22.24 (17)°. The nitrate anions show a pronounced non-parallel orientation, forming an inter­planar angle of 78.4 (1)°.

## 3. Supra­molecular features

**Figure 2:**
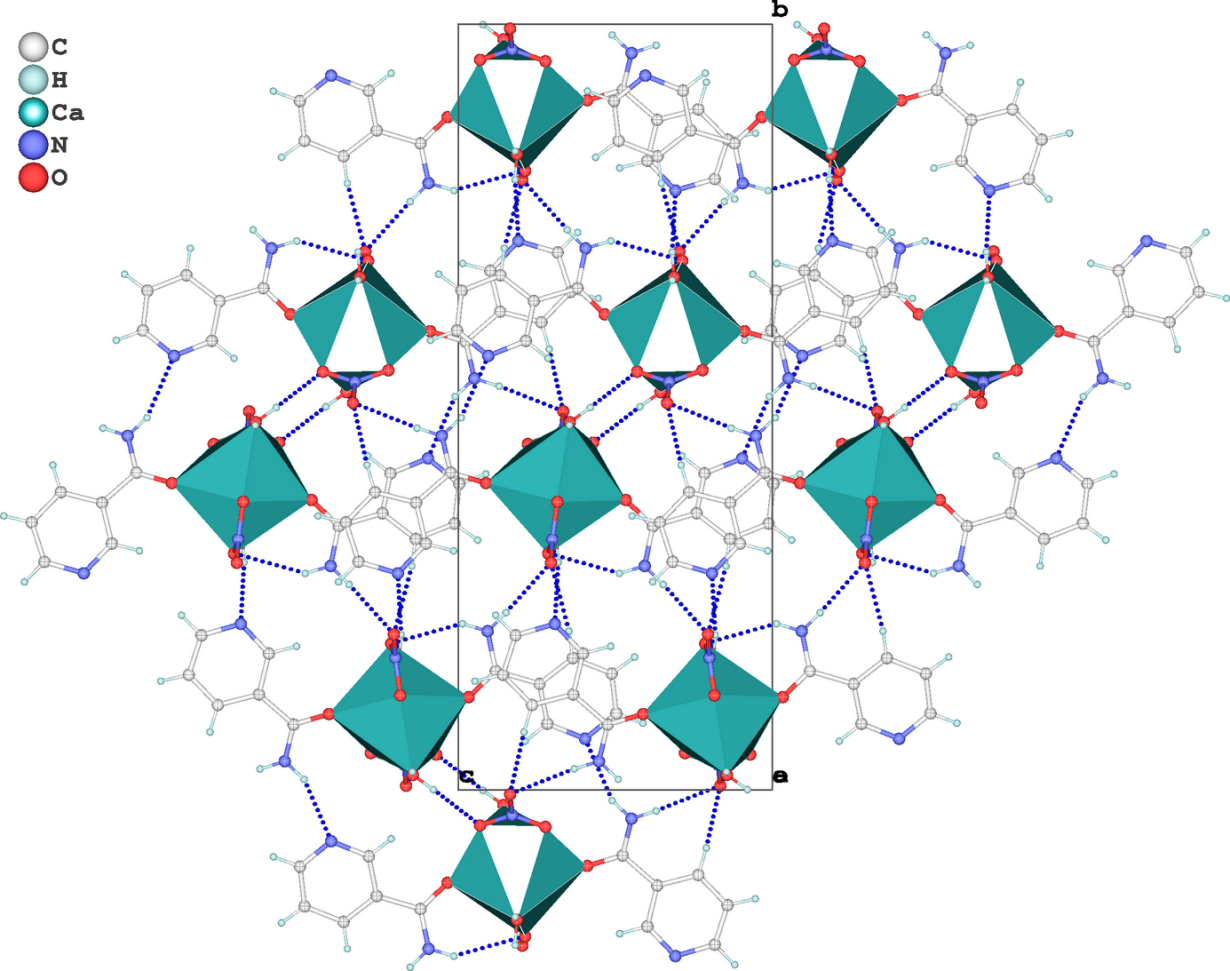
Crystal packing of (**I**) viewed along the *a*-axis direction. Inter­molecular hydrogen bonds are shown as dashed lines.

The supra­molecular architecture of (**I**) is consolidated by a network of hydrogen bonds—including both classical (O—H⋯O, N—H⋯O) and non-classical (C—H⋯O) types—as well as π–π stacking inter­actions, which collectively reinforce the three-dimensional supra­molecular framework. The hydrogen-bonding network involves coordinated water mol­ecules (O1*W* and O2*W*), amide –NH_2_ groups, pyridyl nitro­gen atoms (N1*A*/N1*B*), coordinated nitrate oxygen atoms (O2*A* and O2*B*) and uncoordinated nitrate oxygen atoms (O3*A* and O3*B*) (Table 2 ▸ and Fig. 2 ▸). Propagation of the network along the [100] direction is mediated by O2*W*—H2*WA*⋯O2*A* and N2*B*—H2*BA*⋯N1*A* bonds, related by an inversion center (symmetry operation: 1 − *x*, 1 − *y*, 1 − *z*), while along [001], O1*W*—H1*WB*⋯N1*B* and N2*A*—H2*AB*⋯O3*B* bonds extend the structure *via* a screw axis (−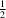 + *x*, 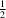 − *y*, −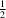 + *z*). Along [010], O2*W*—H2*WB*⋯O3*A* and a weaker C5*B*—H5*B*⋯O3*A* inter­action propagate *via* inversion/translation symmetry (1 − *x*, 1 − *y*, 2 − *z*). The water mol­ecules serve as pivotal hydrogen-bond donors: O1*W* links to O3*B* and N1*B*, while O2*W* donates to O2*A* and O3*A*. The amide groups contribute significantly, with N2*B* donating to N1*A* and O3*A* and N2*A*—H2*AA*⋯O2*B*, forming an intra­molecular contact. The nitrate groups act as hydrogen-bond acceptors: the coordinated oxygen atoms O2*A* and O2*B* engage in classical N—H⋯O and O—H⋯O inter­actions, while the uncoordinated O3*A* and O3*B* atoms participate in multiple contacts (six in total), including a notably linear C5*A*—H5*A*⋯O3*B* inter­action (174°), further reinforcing the structure *via* weak C—H⋯O bonding. Graph-set analysis reveals centrosymmetric 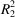
(8) rings from O2*W*—H⋯O2*A* inter­actions; 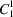
(6) chains along [010] (O2*W*—H2*WB*⋯O3*A*), and two distinct chains along [001]: 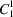
(6) from O1*W*—H1*WA*⋯O3*B* and 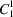
(8) from O1*W*—H1*WB*⋯N1*B*. Higher-order ring motifs include 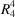
(12), 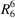
(16), and 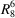
(20), reflecting increasing hydrogen-bonding complexity, with water mol­ecules serving as key structural nodes. In addition to hydrogen bonding, π–π stacking inter­actions are observed between pyridyl rings of nicotinamide ligands, involving centroids *Cg*1 (C1*A*–C5*A*/N1*A*) and *Cg*2 (C1*B*–C5*B*/N1*B*), related by the symmetry operations −1 + *x*, *y*, −1 + *z* and 1 + *x*, *y*, 1 + *z*. These inter­actions feature a centroid-to-centroid distance of 3.783 (2) Å, a dihedral angle of 7.57 (10)°, and a slippage of 1.00–1.17 Å, consistent with a parallel-displaced stacking motif, further consolidating the three-dimensional supra­molecular assembly.

## 4. Hirshfeld surface analysis

**Figure 3:**
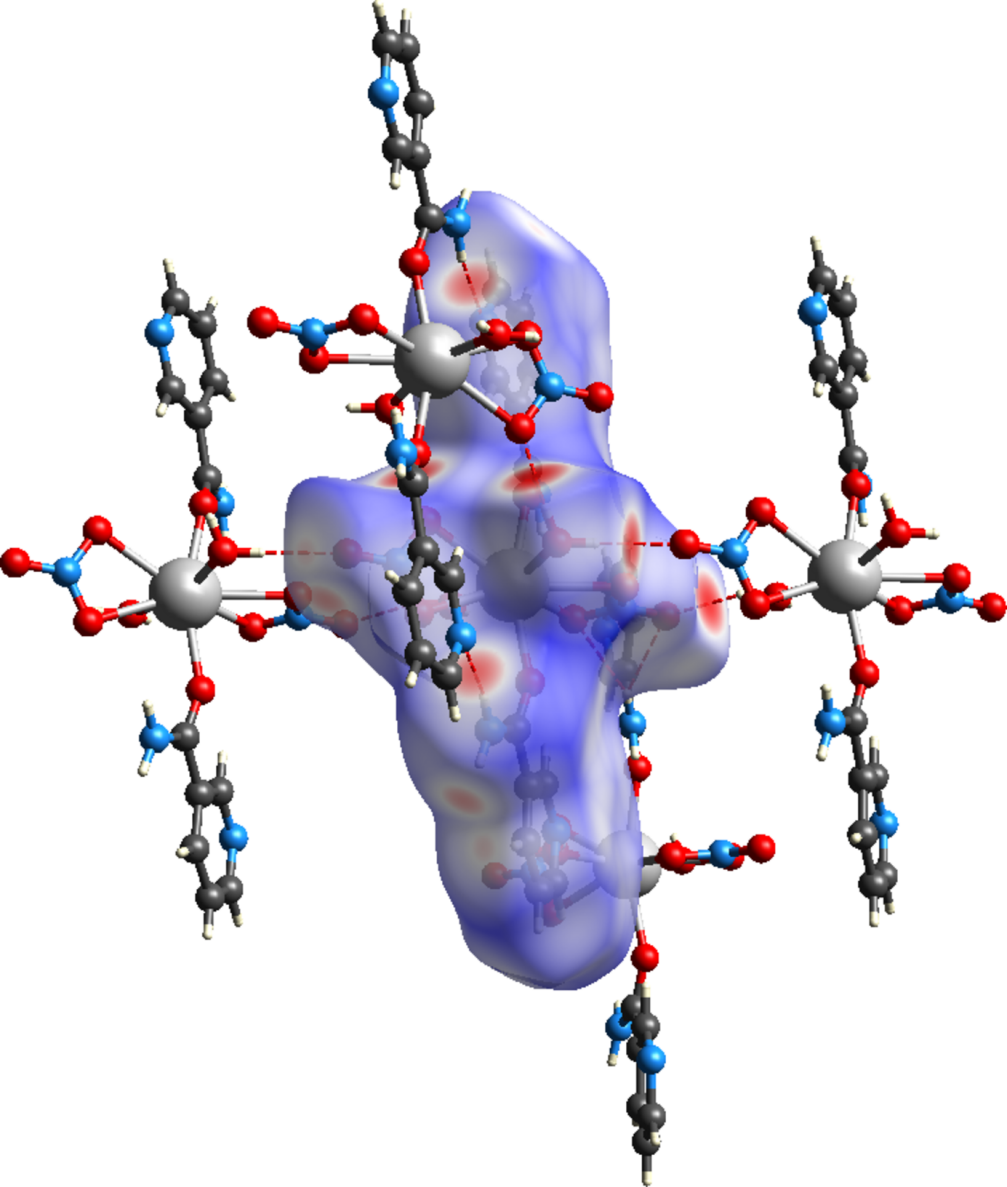
View of the three-dimensional Hirshfeld surface of (**I**) plotted over *d*_norm_. Hydrogen bonds are indicated by red dotted lines.

**Figure 4:**
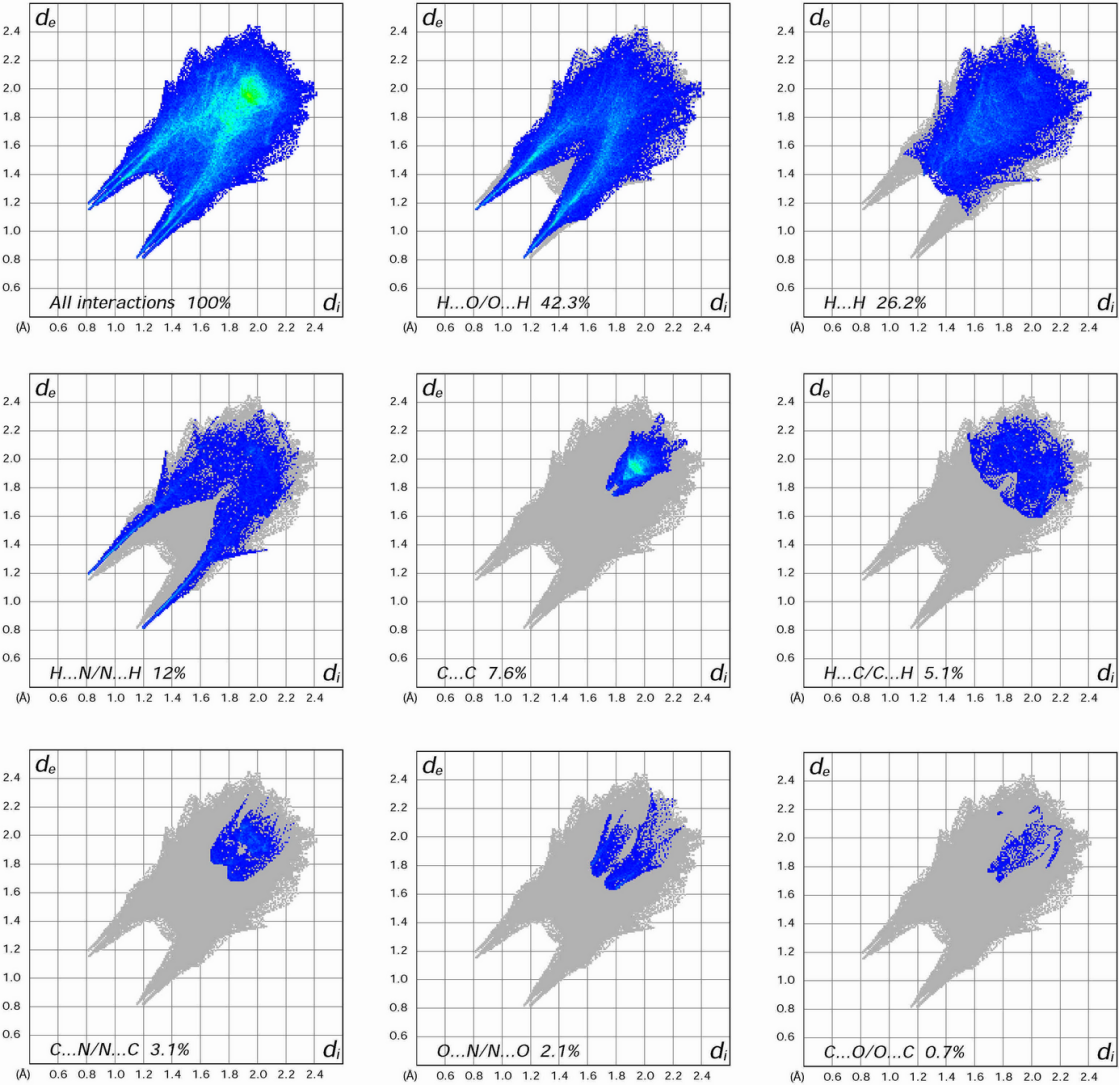
The two-dimensional fingerprint plots for (**I**), showing all inter­actions and different contact types. The *d*_i_ and *d_e_* values represent the closest inter­nal and external distances (in Å) from given points on the Hirshfeld surface.

To further investigate the inter­molecular inter­actions present in the title compound, a Hirshfeld surface analysis was performed using *CrystalExplorer17* (Spackman *et al.*, 2021 ▸), and the corresponding two-dimensional fingerprint plots were generated. The three-dimensional Hirshfeld surface of the complex, mapped over normalized contact distance (*d*_norm_), is shown in Fig. 3 ▸. Intense red spots are clearly visible near atoms O1*W*, O2*W*, O2*A*, and O3*B*, indicating close contacts associated with strong hydrogen bonding. These visual cues correspond well with the short O—H⋯O and N—H⋯O hydrogen bonds identified crystallographically. Qu­anti­tative surface analysis reveal that O⋯H/H⋯O contacts dominate the inter­molecular landscape, contributing 42.3% of the total surface. H⋯H contacts contribute 26.2%, indicative of extensive van der Waals inter­actions (Fig. 4 ▸). Additional contributions are observed from N⋯H/H⋯N (12.0%), C⋯C (7.6%; π–π stacking between aromatic rings), H⋯C/C⋯H (5.1%; weak C—H⋯π and C—H⋯C*sp*^2^ inter­actions), C⋯N/N⋯C (3.1%), N⋯O/O⋯N (2.1%), and C⋯O/O⋯C (0.7%) contacts. The fingerprint plot for O⋯H/H⋯O contacts exhibits a prominent symmetric double-spike pattern, characteristic of directional and geometrically well-matched hydrogen bonds. This pattern reflects nearly equal inter­nal and external contact distances (*d_i_* ≃ *d_e_*), consistent with classical hydrogen-bonding geometry. The symmetry of the spikes also supports the occurrence of bifurcated hydrogen bonding, notably where O1*W* acts as a donor to two acceptors (O2*A*). These inter­actions, in combination with π–π stacking, reinforce the stability and cohesion of the three-dimensional supra­molecular architecture.

## 5. Database survey

A search of the Cambridge Structural Database (CSD, version 6.00, April 2025; Groom *et al.*, 2016 ▸) yielded six calcium(II) complexes featuring nicotinamide ligands. In these structures, the nicotinamide mol­ecule is typically coordinated to the calcium atom *via* its pyridyl nitro­gen atom, while the amide moiety remains non-coordinating. Coordinated water mol­ecules and counter-ions such as nitrate or chloride are present and contribute to the formation of extended supra­molecular networks through hydrogen bonding. On a broader scale, more than 400 crystal structures involving nicotinamide ligands bound to various metal centers have been reported. These complexes frequently exhibit N—H⋯O and O—H⋯O hydrogen bonding inter­actions, and in some cases, π–π stacking between pyridine rings. Notable structurally related calcium–nicotinamide complexes include CSD refcode BAFZER, a pyridine-3-carboxamide derivative featuring extended hydrogen bonding (Song *et al.*, 2020 ▸); KOPBIC and KOPBOI, chain-type and monomeric complexes containing chloride and nicotinamide ligands (Braga *et al.*, 2014 ▸); REZWAW and REZWEA, which feature coordinated water mol­ecule and nicotinamide with chloride counter-ions (Braga *et al.*, 2011 ▸); and YEKHEF, a bis­(pyridine-3-carboxamide) calcium complex incorporating tri­nitro­phenolate ligands (Parsekar *et al.*, 2022 ▸). These structures demonstrate the flexible coordination behavior of nicotinamide and its consistent role in participating in metal–organic assemblies through directional non-covalent inter­actions. In addition, approximately 70 calcium(II) complexes containing nitrate anions are reported in the CSD. In most of these, nitrate acts as a bidentate ligand coordinating in a κ^2^*O*,*O*′ fashion. Bridging coordination modes (μ_2_-κ^2^*O*,*O*′), in which the nitrate anion links two calcium atoms, are also observed. Tridentate coordination (κ^3^*O*,*O*′,*O*′′) is extremely rare. In other structures, nitrate remains as an uncoordinated counter-ion and functions as a hydrogen-bond acceptor in the formation of supra­molecular networks.

## 6. Synthesis and crystallization

The title compound was synthesized by a mechanochemical method using a ball mill operating at 21 Hz. A mixture of calcium nitrate tetra­hydrate (2.3619 g, 0.0100 mol) and nicotinamide (2.4426 g, 0.0200 mol) was ground in a ball mill at room temperature for 9–12 minutes. The product yield was 87.0%. The resulting powder was dissolved in ethanol, and colorless prismatic crystals, stable at room temperature, were obtained by slow evaporation in a vacuum desiccator over a saturated CaCl_2_ solution after 15 days. Suitable single crystals were selected for X-ray diffraction analysis.

## 7. Refinement

Crystal data, data collection and structure refinement details are summarized in Table 3 ▸. Hydrogen atoms bonded to carbon atoms were placed in geometrically idealized positions, with C—H = 0.93 Å and refined using a riding model with *U*_iso_(H) = 1.2*U*_eq_(C). The hydrogen atoms of the coordinated water mol­ecules and the amino groups were located from difference-Fourier maps and refined with restrained geometry (O—H and N—H distances) and displacement parameters.

## Supplementary Material

Crystal structure: contains datablock(s) I. DOI: 10.1107/S2056989025006759/hb8147sup1.cif

Structure factors: contains datablock(s) I. DOI: 10.1107/S2056989025006759/hb8147Isup2.hkl

CCDC reference: 2476945

Additional supporting information:  crystallographic information; 3D view; checkCIF report

## Figures and Tables

**Table 1:** Selected bond lengths (Å)

Ca1—O4*B*	2.5825 (18)	Ca1—O1*B*	2.3150 (16)
Ca1—O2*W*	2.3820 (16)	Ca1—O1*A*	2.3359 (15)
Ca1—O2*B*	2.5752 (17)	Ca1—O4*A*	2.5362 (19)
Ca1—O1*W*	2.3459 (18)	Ca1—O2*A*	2.5537 (18)

**Table 2:** Hydrogen-bond geometry (Å, °)

*D*—H⋯*A*	*D*—H	H⋯*A*	*D*⋯*A*	*D*—H⋯*A*
N2*A*—H2*AB*⋯O3*B*^i^	0.85 (1)	2.42 (2)	3.180 (3)	149 (3)
N2*A*—H2*AA*⋯O2*B*	0.86 (1)	2.53 (2)	3.284 (3)	146 (3)
N2*B*—H2*BA*⋯N1*A*^ii^	0.86 (1)	2.21 (2)	3.024 (3)	157 (3)
N2*B*—H2*BB*⋯O3*A*^iii^	0.86 (1)	2.35 (1)	3.211 (3)	174 (3)
O1*W*—H1*WA*⋯O3*B*^iv^	0.84 (1)	2.04 (2)	2.761 (3)	144 (3)
O1*W*—H1*WB*⋯N1*B*^i^	0.85 (1)	2.00 (1)	2.833 (3)	164 (3)
O2*W*—H2*WA*⋯O2*A*^ii^	0.85 (1)	1.97 (1)	2.813 (2)	178 (3)
O2*W*—H2*WB*⋯O3*A*^v^	0.84 (1)	2.10 (2)	2.904 (3)	160 (4)
C5*A*—H5*A*⋯O3*B*^i^	0.93	2.41	3.336 (3)	174
C5*B*—H5*B*⋯O3*A*^iii^	0.93	2.49	3.362 (3)	155

Symmetry codes: (i) 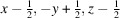; (ii) 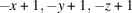; (iii) 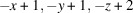; (iv) 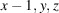; (v) 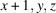.

**Table 3:** Experimental details

Crystal data	
Chemical formula	[Ca(NO_3_)_2_(C_6_H_6_N_2_O)_2_(H_2_O)_2_]
*M*_r_	444.39
Crystal system, space group	Monoclinic, *P*2_1_/*n*
Temperature (K)	292
*a*, *b*, *c* (Å)	7.5454 (3), 24.8759 (9), 10.7807 (4)
β (°)	108.777 (4)
*V* (Å^3^)	1915.83 (13)
*Z*	4
Radiation type	Cu *K*α
μ (mm^−1^)	3.44
Crystal size (mm)	0.3 × 0.2 × 0.15

Data collection	
Diffractometer	Xcalibur, Ruby
Absorption correction	Multi-scan (*CrysAlis PRO*; Rigaku OD, 2022 ▸).
*T*_min_, *T*_max_	0.695, 1.000
No. of measured, independent and observed [*I* > 2σ(*I*)] reflections	7612, 3862, 3249
*R*_int_	0.029
(sin θ/λ)_max_ (Å^−1^)	0.630

Refinement	
*R*[*F*^2^ > 2σ(*F*^2^)], *wR*(*F*^2^), *S*	0.041, 0.114, 1.03
No. of reflections	3862
No. of parameters	295
No. of restraints	8
H-atom treatment	H atoms treated by a mixture of independent and constrained refinement
Δρ_max_, Δρ_min_ (e Å^−3^)	0.28, −0.22

Computer programs: *CrysAlis PRO* (Rigaku OD, 2022 ▸), *SHELXT2018/2* (Sheldrick, 2015*a* ▸), *SHELXL2019/3* (Sheldrick, 2015*b* ▸), *OLEX2* (Dolomanov *et al.*, 2009 ▸) and *publCIF* (Westrip, 2010 ▸).

## supplementary crystallographic information

### Diaquabis(nicotinamide-κO)bis(nitrato-κ2O,O')calcium(II) Crystal data

**Table:**

[Ca(NO_3_)_2_(C_6_H_6_N_2_O)_2_(H_2_O)_2_]	*F*(000) = 920
*M**_r_* = 444.39	*D*_x_ = 1.541 Mg m^−^^3^
Monoclinic, *P*2_1_/*n*	Cu *K*α radiation, λ = 1.54184 Å
*a* = 7.5454 (3) Å	Cell parameters from 3138 reflections
*b* = 24.8759 (9) Å	θ = 4.3–75.6°
*c* = 10.7807 (4) Å	µ = 3.44 mm^−^^1^
β = 108.777 (4)°	*T* = 292 K
*V* = 1915.83 (13) Å^3^	Block, colourless
*Z* = 4	0.3 × 0.2 × 0.15 mm

### Diaquabis(nicotinamide-κO)bis(nitrato-κ2O,O')calcium(II) Data collection

**Table:**

Xcalibur, Ruby diffractometer	3862 independent reflections
Radiation source: Enhance (Cu) X-ray Source	3249 reflections with *I* > 2σ(*I*)
Graphite monochromator	*R*_int_ = 0.029
Detector resolution: 10.2576 pixels mm^-1^	θ_max_ = 76.1°, θ_min_ = 3.6°
ω scans	*h* = −9→4
Absorption correction: multi-scan (CrysAlisPro; Rigaku OD, 2022).	*k* = −21→30
*T*_min_ = 0.695, *T*_max_ = 1.000	*l* = −12→13
7612 measured reflections	

### Diaquabis(nicotinamide-κO)bis(nitrato-κ2O,O')calcium(II) Refinement

**Table:**

Refinement on *F*^2^	Hydrogen site location: mixed
Least-squares matrix: full	H atoms treated by a mixture of independent and constrained refinement
*R*[*F*^2^ > 2σ(*F*^2^)] = 0.041	*w* = 1/[σ^2^(*F*_o_^2^) + (0.0628*P*)^2^ + 0.3395*P*] where *P* = (*F*_o_^2^ + 2*F*_c_^2^)/3
*wR*(*F*^2^) = 0.114	(Δ/σ)_max_ < 0.001
*S* = 1.03	Δρ_max_ = 0.28 e Å^−^^3^
3862 reflections	Δρ_min_ = −0.22 e Å^−^^3^
295 parameters	Extinction correction: *SHELXL2019/3* (Sheldrick, 2015b), Fc^*^=kFc[1+0.001xFc^2^λ^3^/sin(2θ)]^-1/4^
8 restraints	Extinction coefficient: 0.0024 (3)
Primary atom site location: dual	

### Diaquabis(nicotinamide-κO)bis(nitrato-κ2O,O')calcium(II) Special details

**Table:**

Geometry. All esds (except the esd in the dihedral angle between two l.s. planes) are estimated using the full covariance matrix. The cell esds are taken into account individually in the estimation of esds in distances, angles and torsion angles; correlations between esds in cell parameters are only used when they are defined by crystal symmetry. An approximate (isotropic) treatment of cell esds is used for estimating esds involving l.s. planes.

### Diaquabis(nicotinamide-κO)bis(nitrato-κ2O,O')calcium(II) Fractional atomic coordinates and isotropic or equivalent isotropic displacement parameters (Å^2^)

**Table:**

	*x*	*y*	*z*	*U*_iso_*/*U*_eq_	
Ca1	0.67003 (6)	0.39970 (2)	0.68777 (4)	0.03229 (14)	
O4B	1.0018 (2)	0.37641 (6)	0.68407 (17)	0.0493 (4)	
O2W	0.8098 (2)	0.48033 (6)	0.64428 (17)	0.0468 (4)	
O2B	0.8417 (2)	0.30884 (6)	0.71333 (18)	0.0499 (4)	
O1W	0.4593 (3)	0.33030 (7)	0.6873 (2)	0.0579 (5)	
O1B	0.8260 (3)	0.40080 (7)	0.91112 (15)	0.0547 (4)	
N3B	0.9890 (2)	0.32726 (7)	0.70013 (18)	0.0392 (4)	
O1A	0.6075 (3)	0.37876 (7)	0.46656 (15)	0.0527 (4)	
O4A	0.4497 (3)	0.45182 (8)	0.77806 (17)	0.0599 (5)	
O3A	0.2005 (2)	0.49445 (8)	0.6654 (2)	0.0627 (5)	
O2A	0.3761 (3)	0.45368 (8)	0.56996 (17)	0.0592 (5)	
N3A	0.3399 (3)	0.46709 (7)	0.6718 (2)	0.0427 (4)	
O3B	1.1193 (2)	0.29668 (7)	0.7045 (2)	0.0696 (6)	
N2B	0.7909 (4)	0.46252 (9)	1.0519 (2)	0.0610 (6)	
C6B	0.8402 (3)	0.41447 (9)	1.0239 (2)	0.0403 (5)	
N1A	0.3231 (3)	0.43303 (9)	0.0959 (2)	0.0570 (5)	
C1B	0.9154 (3)	0.37523 (8)	1.1338 (2)	0.0368 (4)	
N2A	0.5831 (4)	0.29261 (9)	0.4060 (2)	0.0690 (7)	
N1B	0.9965 (3)	0.28306 (8)	1.1918 (3)	0.0623 (6)	
C1A	0.4714 (3)	0.36137 (8)	0.24086 (19)	0.0363 (4)	
C2A	0.4057 (4)	0.41346 (9)	0.2157 (2)	0.0475 (5)	
H2A	0.420138	0.436219	0.286721	0.057*	
C6A	0.5592 (3)	0.34461 (9)	0.3802 (2)	0.0410 (5)	
C2B	0.9309 (4)	0.32181 (9)	1.1026 (3)	0.0482 (5)	
H2B	0.893628	0.312300	1.014492	0.058*	
C5A	0.4520 (4)	0.32777 (10)	0.1350 (2)	0.0515 (6)	
H5A	0.494550	0.292461	0.147272	0.062*	
C5B	0.9718 (4)	0.38893 (9)	1.2648 (2)	0.0513 (6)	
H5B	0.963082	0.424312	1.290129	0.062*	
C3A	0.3056 (4)	0.39995 (11)	−0.0036 (2)	0.0563 (6)	
H3A	0.247755	0.412757	−0.088068	0.068*	
C4A	0.3681 (4)	0.34791 (11)	0.0112 (2)	0.0620 (7)	
H4A	0.354019	0.326380	−0.061917	0.074*	
C4B	1.0411 (5)	0.34951 (12)	1.3576 (3)	0.0655 (8)	
H4B	1.080955	0.357922	1.446329	0.079*	
C3B	1.0502 (4)	0.29799 (12)	1.3170 (3)	0.0657 (8)	
H3B	1.096600	0.271718	1.380608	0.079*	
H2BA	0.754 (4)	0.4861 (9)	0.990 (2)	0.067 (9)*	
H2AA	0.632 (4)	0.2829 (13)	0.4866 (14)	0.077 (10)*	
H2BB	0.800 (5)	0.4726 (14)	1.1305 (16)	0.083 (11)*	
H2AB	0.547 (5)	0.2684 (10)	0.347 (2)	0.079 (10)*	
H2WA	0.751 (4)	0.4995 (10)	0.5789 (19)	0.059 (8)*	
H1WA	0.3466 (18)	0.3343 (13)	0.682 (3)	0.068 (9)*	
H1WB	0.486 (4)	0.2973 (5)	0.682 (3)	0.069 (9)*	
H2WB	0.921 (2)	0.4763 (19)	0.646 (4)	0.118 (16)*	

### Diaquabis(nicotinamide-κO)bis(nitrato-κ2O,O')calcium(II) Atomic displacement parameters (Å^2^)

**Table:**

	*U*^11^	*U*^22^	*U*^33^	*U*^12^	*U*^13^	*U*^23^
Ca1	0.0386 (2)	0.0279 (2)	0.0297 (2)	0.00102 (16)	0.01011 (16)	−0.00019 (14)
O4B	0.0591 (10)	0.0293 (7)	0.0599 (10)	−0.0019 (7)	0.0198 (8)	0.0047 (7)
O2W	0.0478 (9)	0.0351 (8)	0.0556 (10)	0.0002 (7)	0.0139 (8)	0.0072 (7)
O2B	0.0421 (8)	0.0426 (8)	0.0689 (11)	−0.0012 (7)	0.0235 (8)	0.0038 (8)
O1W	0.0513 (10)	0.0392 (9)	0.0926 (14)	−0.0053 (8)	0.0362 (10)	0.0000 (9)
O1B	0.0630 (11)	0.0628 (11)	0.0319 (8)	0.0128 (9)	0.0065 (7)	−0.0021 (7)
N3B	0.0393 (9)	0.0327 (9)	0.0436 (9)	0.0008 (7)	0.0106 (7)	−0.0003 (7)
O1A	0.0720 (11)	0.0480 (9)	0.0350 (8)	−0.0019 (9)	0.0129 (7)	−0.0094 (7)
O4A	0.0649 (11)	0.0678 (12)	0.0464 (9)	0.0157 (10)	0.0171 (8)	0.0025 (9)
O3A	0.0501 (10)	0.0502 (10)	0.0915 (14)	0.0139 (8)	0.0279 (10)	−0.0030 (9)
O2A	0.0686 (11)	0.0631 (11)	0.0467 (9)	0.0261 (9)	0.0197 (8)	0.0074 (8)
N3A	0.0432 (10)	0.0321 (9)	0.0556 (11)	0.0026 (8)	0.0200 (9)	−0.0001 (8)
O3B	0.0448 (9)	0.0398 (9)	0.1299 (19)	0.0071 (8)	0.0361 (11)	0.0030 (10)
N2B	0.0946 (18)	0.0422 (11)	0.0405 (11)	0.0214 (12)	0.0138 (12)	0.0064 (9)
C6B	0.0443 (12)	0.0404 (11)	0.0321 (9)	0.0045 (9)	0.0065 (9)	0.0005 (8)
N1A	0.0712 (14)	0.0459 (11)	0.0516 (12)	0.0029 (11)	0.0168 (11)	0.0109 (9)
C1B	0.0382 (10)	0.0347 (10)	0.0361 (10)	0.0017 (9)	0.0098 (8)	0.0009 (8)
N2A	0.117 (2)	0.0409 (12)	0.0364 (11)	0.0119 (13)	0.0067 (12)	−0.0006 (9)
N1B	0.0690 (15)	0.0347 (10)	0.0839 (17)	0.0078 (10)	0.0255 (13)	0.0071 (11)
C1A	0.0423 (11)	0.0335 (10)	0.0332 (10)	−0.0040 (9)	0.0124 (8)	−0.0032 (8)
C2A	0.0607 (14)	0.0370 (11)	0.0447 (12)	0.0021 (10)	0.0167 (11)	−0.0025 (9)
C6A	0.0492 (12)	0.0394 (11)	0.0334 (10)	0.0011 (9)	0.0121 (9)	−0.0048 (9)
C2B	0.0536 (13)	0.0367 (11)	0.0543 (13)	0.0052 (10)	0.0176 (11)	−0.0036 (10)
C5A	0.0749 (16)	0.0399 (12)	0.0388 (11)	0.0007 (12)	0.0172 (11)	−0.0051 (9)
C5B	0.0733 (17)	0.0381 (12)	0.0383 (11)	0.0047 (11)	0.0121 (11)	0.0007 (9)
C3A	0.0656 (16)	0.0602 (16)	0.0404 (12)	−0.0086 (13)	0.0132 (11)	0.0098 (11)
C4A	0.091 (2)	0.0587 (16)	0.0339 (11)	−0.0082 (15)	0.0167 (12)	−0.0085 (11)
C4B	0.089 (2)	0.0599 (16)	0.0414 (13)	0.0072 (15)	0.0130 (13)	0.0135 (12)
C3B	0.0740 (18)	0.0491 (15)	0.0721 (19)	0.0097 (14)	0.0211 (15)	0.0280 (14)

### Diaquabis(nicotinamide-κO)bis(nitrato-κ2O,O')calcium(II) Geometric parameters (Å, º)

**Table:**

Ca1—O4B	2.5825 (18)	N1A—C2A	1.333 (3)
Ca1—O2W	2.3820 (16)	N1A—C3A	1.325 (3)
Ca1—O2B	2.5752 (17)	C1B—C2B	1.385 (3)
Ca1—O1W	2.3459 (18)	C1B—C5B	1.381 (3)
Ca1—O1B	2.3150 (16)	N2A—C6A	1.323 (3)
Ca1—O1A	2.3359 (15)	N2A—H2AA	0.863 (10)
Ca1—O4A	2.5362 (19)	N2A—H2AB	0.852 (10)
Ca1—O2A	2.5537 (18)	N1B—C2B	1.339 (3)
O4B—N3B	1.243 (2)	N1B—C3B	1.332 (4)
O2W—H2WA	0.848 (10)	C1A—C2A	1.383 (3)
O2W—H2WB	0.844 (10)	C1A—C6A	1.493 (3)
O2B—N3B	1.252 (2)	C1A—C5A	1.384 (3)
O1W—H1WA	0.839 (10)	C2A—H2A	0.9300
O1W—H1WB	0.852 (10)	C2B—H2B	0.9300
O1B—C6B	1.234 (3)	C5A—H5A	0.9300
N3B—O3B	1.232 (2)	C5A—C4A	1.374 (3)
O1A—C6A	1.226 (3)	C5B—H5B	0.9300
O4A—N3A	1.238 (3)	C5B—C4B	1.378 (3)
O3A—N3A	1.236 (2)	C3A—H3A	0.9300
O2A—N3A	1.258 (2)	C3A—C4A	1.370 (4)
N2B—C6B	1.315 (3)	C4A—H4A	0.9300
N2B—H2BA	0.862 (10)	C4B—H4B	0.9300
N2B—H2BB	0.864 (10)	C4B—C3B	1.363 (4)
C6B—C1B	1.498 (3)	C3B—H3B	0.9300
			
O2W—Ca1—O4B	72.13 (5)	O3A—N3A—O2A	121.1 (2)
O2W—Ca1—O2B	121.39 (6)	C6B—N2B—H2BA	119 (2)
O2W—Ca1—O4A	91.80 (6)	C6B—N2B—H2BB	123 (2)
O2W—Ca1—O2A	80.09 (6)	H2BA—N2B—H2BB	117 (3)
O2B—Ca1—O4B	49.31 (5)	O1B—C6B—N2B	122.4 (2)
O1W—Ca1—O4B	119.65 (6)	O1B—C6B—C1B	119.3 (2)
O1W—Ca1—O2W	164.15 (7)	N2B—C6B—C1B	118.29 (19)
O1W—Ca1—O2B	70.83 (6)	C3A—N1A—C2A	116.8 (2)
O1W—Ca1—O4A	81.35 (7)	C2B—C1B—C6B	118.3 (2)
O1W—Ca1—O2A	84.63 (7)	C5B—C1B—C6B	124.2 (2)
O1B—Ca1—O4B	81.25 (6)	C5B—C1B—C2B	117.6 (2)
O1B—Ca1—O2W	94.91 (7)	C6A—N2A—H2AA	118 (2)
O1B—Ca1—O2B	80.17 (6)	C6A—N2A—H2AB	123 (2)
O1B—Ca1—O1W	97.38 (7)	H2AA—N2A—H2AB	119 (3)
O1B—Ca1—O1A	158.82 (7)	C3B—N1B—C2B	116.7 (2)
O1B—Ca1—O4A	76.78 (6)	C2A—C1A—C6A	118.31 (19)
O1B—Ca1—O2A	125.48 (6)	C2A—C1A—C5A	117.9 (2)
O1A—Ca1—O4B	79.19 (6)	C5A—C1A—C6A	123.8 (2)
O1A—Ca1—O2W	86.77 (6)	N1A—C2A—C1A	124.0 (2)
O1A—Ca1—O2B	80.96 (6)	N1A—C2A—H2A	118.0
O1A—Ca1—O1W	85.39 (7)	C1A—C2A—H2A	118.0
O1A—Ca1—O4A	124.32 (6)	O1A—C6A—N2A	122.0 (2)
O1A—Ca1—O2A	75.63 (6)	O1A—C6A—C1A	119.9 (2)
O4A—Ca1—O4B	151.52 (6)	N2A—C6A—C1A	118.10 (19)
O4A—Ca1—O2B	140.96 (6)	C1B—C2B—H2B	118.1
O4A—Ca1—O2A	49.50 (5)	N1B—C2B—C1B	123.9 (2)
O2A—Ca1—O4B	143.26 (6)	N1B—C2B—H2B	118.1
O2A—Ca1—O2B	147.25 (6)	C1A—C5A—H5A	120.8
N3B—O4B—Ca1	95.69 (12)	C4A—C5A—C1A	118.3 (2)
Ca1—O2W—H2WA	119 (2)	C4A—C5A—H5A	120.8
Ca1—O2W—H2WB	113 (3)	C1B—C5B—H5B	120.4
H2WA—O2W—H2WB	109 (4)	C4B—C5B—C1B	119.1 (2)
N3B—O2B—Ca1	95.80 (12)	C4B—C5B—H5B	120.4
Ca1—O1W—H1WA	126 (2)	N1A—C3A—H3A	118.2
Ca1—O1W—H1WB	122 (2)	N1A—C3A—C4A	123.5 (2)
H1WA—O1W—H1WB	112 (3)	C4A—C3A—H3A	118.2
C6B—O1B—Ca1	151.91 (16)	C5A—C4A—H4A	120.3
O4B—N3B—O2B	119.18 (18)	C3A—C4A—C5A	119.4 (2)
O3B—N3B—O4B	121.04 (19)	C3A—C4A—H4A	120.3
O3B—N3B—O2B	119.77 (18)	C5B—C4B—H4B	120.6
C6A—O1A—Ca1	147.46 (16)	C3B—C4B—C5B	118.9 (3)
N3A—O4A—Ca1	97.34 (13)	C3B—C4B—H4B	120.6
N3A—O2A—Ca1	95.91 (13)	N1B—C3B—C4B	123.8 (2)
O4A—N3A—O2A	117.23 (19)	N1B—C3B—H3B	118.1
O3A—N3A—O4A	121.7 (2)	C4B—C3B—H3B	118.1
			
Ca1—O4B—N3B—O2B	−1.3 (2)	N1A—C3A—C4A—C5A	0.8 (5)
Ca1—O4B—N3B—O3B	179.5 (2)	C1B—C5B—C4B—C3B	−0.6 (5)
Ca1—O2B—N3B—O4B	1.3 (2)	C1A—C5A—C4A—C3A	−0.5 (4)
Ca1—O2B—N3B—O3B	−179.44 (19)	C2A—N1A—C3A—C4A	−0.3 (4)
Ca1—O1B—C6B—N2B	−38.9 (5)	C2A—C1A—C6A—O1A	−15.3 (3)
Ca1—O1B—C6B—C1B	140.9 (3)	C2A—C1A—C6A—N2A	164.9 (3)
Ca1—O1A—C6A—N2A	−25.1 (5)	C2A—C1A—C5A—C4A	−0.2 (4)
Ca1—O1A—C6A—C1A	155.2 (2)	C6A—C1A—C2A—N1A	−179.3 (2)
Ca1—O4A—N3A—O3A	178.72 (18)	C6A—C1A—C5A—C4A	179.9 (2)
Ca1—O4A—N3A—O2A	−1.4 (2)	C2B—C1B—C5B—C4B	0.4 (4)
Ca1—O2A—N3A—O4A	1.4 (2)	C2B—N1B—C3B—C4B	0.2 (5)
Ca1—O2A—N3A—O3A	−178.73 (18)	C5A—C1A—C2A—N1A	0.8 (4)
O1B—C6B—C1B—C2B	−12.9 (3)	C5A—C1A—C6A—O1A	164.6 (2)
O1B—C6B—C1B—C5B	166.7 (2)	C5A—C1A—C6A—N2A	−15.2 (4)
N2B—C6B—C1B—C2B	166.9 (2)	C5B—C1B—C2B—N1B	0.2 (4)
N2B—C6B—C1B—C5B	−13.6 (4)	C5B—C4B—C3B—N1B	0.3 (5)
C6B—C1B—C2B—N1B	179.8 (2)	C3A—N1A—C2A—C1A	−0.5 (4)
C6B—C1B—C5B—C4B	−179.2 (2)	C3B—N1B—C2B—C1B	−0.5 (4)

### Diaquabis(nicotinamide-κO)bis(nitrato-κ2O,O')calcium(II) Hydrogen-bond geometry (Å, º)

**Table:**

*D*—H···*A*	*D*—H	H···*A*	*D*···*A*	*D*—H···*A*
N2*A*—H2*AB*···O3*B*^i^	0.85 (1)	2.42 (2)	3.180 (3)	149 (3)
N2*A*—H2*AA*···O2*B*	0.86 (1)	2.53 (2)	3.284 (3)	146 (3)
N2*B*—H2*BA*···N1*A*^ii^	0.86 (1)	2.21 (2)	3.024 (3)	157 (3)
N2*B*—H2*BB*···O3*A*^iii^	0.86 (1)	2.35 (1)	3.211 (3)	174 (3)
O1*W*—H1*WA*···O3*B*^iv^	0.84 (1)	2.04 (2)	2.761 (3)	144 (3)
O1*W*—H1*WB*···N1*B*^i^	0.85 (1)	2.00 (1)	2.833 (3)	164 (3)
O2*W*—H2*WA*···O2*A*^ii^	0.85 (1)	1.97 (1)	2.813 (2)	178 (3)
O2*W*—H2*WB*···O3*A*^v^	0.84 (1)	2.10 (2)	2.904 (3)	160 (4)
C5*A*—H5*A*···O3*B*^i^	0.93	2.41	3.336 (3)	174
C5*B*—H5*B*···O3*A*^iii^	0.93	2.49	3.362 (3)	155

Symmetry codes: (i) *x*−1/2, −*y*+1/2, *z*−1/2; (ii) −*x*+1, −*y*+1, −*z*+1; (iii) −*x*+1, −*y*+1, −*z*+2; (iv) *x*−1, *y*, *z*; (v) *x*+1, *y*, *z*.
